# Toxicological Profile and Anti-Inflammatory Effect of Mucoadhesive Gel from Residues of *Agave sisalana* and *Punica granatum*

**DOI:** 10.3390/gels9120942

**Published:** 2023-11-30

**Authors:** Júlia Amanda Rodrigues Fracasso, Ingrid Yuri Galindo Sikina, Luísa Taynara Silvério da Costa, Lucas Pires Guarnier, João Tadeu Ribeiro-Paes, Fernando Yutaka de Ferreira, Luan Victor Coelho de Almeida, Beatriz de Castro Silva, Débora de Barros Barbosa, Valdecir Farias Ximenes, Desirre Venkli, Amanda Martins Viel, Lucinéia dos Santos

**Affiliations:** 1 School of Dentistry, São Paulo State University (UNESP), 1193, José Bonifacio Street, Araçatuba 16015-050, Brazil; luisatayanara@gmail.com (L.T.S.d.C.); luan.coelho@unesp.br (L.V.C.d.A.); beatriz.c.silva@unesp.br (B.d.C.S.); debora.b.barbosa@unesp.br (D.d.B.B.); lucineia.santos@unesp.br (L.d.S.); 2 Department of Biotechnology, School of Sciences and Languages, São Paulo State University (UNESP), 2100, Dom Antonio Avenue, Assis 19806-900, Brazil; ingrid.yuri@unesp.br (I.Y.G.S.); fernando.yutaka@unesp.br (F.Y.d.F.); 3 Department of Genetics, Ribeirão Preto Medical School, University of São Paulo, Ribeirão Preto 14048-900, Brazil; lucas.guarnier@usp.br (L.P.G.); ribeiro-paes@unesp.br (J.T.R.-P.); 4 Department of Chemistry, Faculty of Sciences, São Paulo State University (UNESP), Bauru 17033-360, Brazil; valdecir.ximenes@unesp.br; 5 Department of Biochemistry and Chemical Technology, Institute of Chemistry, São Paulo State University (UNESP), Araraquara 14800-060, Brazil; 6 São Camilo University Center, São Paulo 04263-200, Brazil; amanda.mviel@hotmail.com; 7 Nossa Senhora do Patrocínio University Center (CEUNSP), Itu 13300-200, Brazil

**Keywords:** herbal, inflammation, saponins

## Abstract

Inflammation is a natural protective reaction of the body against endogenous and exogenous damage, such as tissue injuries, trauma, and infections. Thus, when the response is adequate, inflammation becomes a defense mechanism to repair damaged tissue, whereas when the response is inadequate and persistent, the increase in inflammatory cells, cytosines, and chymosins impair tissue regeneration and promote a response harmful to the organism. One example is chronic tissue inflammation, in which a simple lesion can progress to ulcers and even necrosis. In this situation, the anti-inflammatory medications available in therapy are not always effective. For this reason, the search for new treatments, developed from medicinal plants, has increased. In this direction, the plants *Agave sisalana* (sisal) and *Punica granatum* (pomegranate) are rich in saponins, which are secondary metabolites known for their therapeutic properties, including anti-inflammatory effects. Although Brazil is the world’s leading sisal producer, approximately 95% of the leaves are discarded after fiber extraction. Similarly, pomegranate peel waste is abundant in Brazil. To address the need for safe and effective anti-inflammatory treatments, this study aimed to create a topical mucoadhesive gel containing a combination of sisal (RS) and pomegranate residue (PR) extracts. In vitro experiments examined isolated and combined extracts, as well as the resulting formulation, focusing on (1) a phytochemical analysis (total saponin content); (2) cytotoxicity (MTT assay); and (3) a pharmacological assessment of anti-inflammatory activity (phagocytosis, macrophage spreading, and membrane stability). The results revealed saponin concentrations in grams per 100 g of dry extract as follows: SR—29.91 ± 0.33, PR—15.83 ± 0.93, association (A)—22.99 ± 0.01, base gel (G1)—0.00 ± 0.00, and association gel (G2)—0.52 ± 0.05. In MTT tests for isolated extracts, cytotoxicity values (µg/mL) were 3757.00 for SR and 2064.91 for PR. Conversely, A and G2 exhibited no cytotoxicity, with increased cell viability over time. All three anti-inflammatory tests confirmed the presence of this activity in SR, PR, and A. Notably, G2 demonstrated an anti-inflammatory effect comparable to dexamethasone. In conclusion, the gel containing SR and PR (i.e., A) holds promise as a novel herbal anti-inflammatory treatment. Its development could yield economic, social, and environmental benefits by utilizing discarded materials in Brazil.

## 1. Introduction

Inflammation is a natural protective reaction of an organism against endogenous and exogenous damage (e.g., tissue damage, traumas, infections and immunological reactions), composed of “five pillars”: swelling, heat, redness, pain, and loss of function [1,2,3]. Thus, inflammation is also present in the healing process of injured tissue. This process consists of a perfect and coordinated cascade of cellular, molecular, and biochemical events that interact so that tissue reconstitution occurs. The healing mechanisms are classified into three phases: inflammatory phase, proliferation or granulation phase, and remodeling or maturation phase [4].

Tissue injury, the initial stimulus for the healing process, puts blood elements in contact with collagen and other substances in the extracellular matrix, causing platelet degranulation and activation of the coagulation and complement cascades. This releases several vasoactive and chemotactic mediators that drive the healing process by attracting inflammatory cells to the wound region [1]. Many types of cell from the immunological system actively participate in inflammatory processes, with macrophages being one of these types of cell [4,5,6]. Known as phagocytes, this type of cell is deeply related to innate immunity and, in association with other leukocytes, produces chemical mediators that promote the inflammatory cascade [7].

However, several factors can negatively influence healing by prolonging the inflammatory phase, decreasing fibroblast synthesis and proliferation, angiogenesis, or the synthesis of collagen and proteoglycans. Thus, epidemiological studies reveal that a substantial number of patients with inflammatory skin conditions such as dermatitis, psoriasis, and chronic wounds are at risk of developing necrotic ulcers, particularly when inflammation is inadequately controlled [8,9]. In other words, currently, cutaneous inflammation is one of the most significant global issues, and the subsequent development of necrotic ulcers poses a significant global challenge, affecting millions of individuals each year [1,2].

Furthermore, the resistance to traditional anti-inflammatories, such as non-steroidal anti-inflammatory drugs (NSAIDs), has been observed in a considerable number of cases, making the treatment of these conditions even more challenging [2]. This underscores the urgent need for research and the development of effective anti-inflammatory therapies, as well as strategies to enhance the body’s response to available treatments, in order to mitigate the devastating impact of cutaneous inflammation and reduce the incidence of necrotic ulcers [3,4].

Pharmacological studies that make use of medicinal plants are a prime example of research related to finding alternative drugs that present not only effective anti-inflammatory activity, but an absence of the adverse effects shown by AINEs. The therapeutic effects of plants can be attributed to many secondary metabolites present in plant species. Among these metabolites, saponins stand out, with hemolytic, molluscicides, anti-helminthic, spermicide, hypocholesterolemic, and anti-inflammatory activities. Furthermore, steroidal saponins are used by the industry as a base for corticosteroid anti-inflammatory drugs, while saponin-rich plants are used in traditional medicine for inflammation treatment [5,6,7].

*Agave sisalana* (also known as sisal) and *Punica granatum* (also known as pomegranate) are two examples of many saponin-rich species that can be found in Brazil. Brazil is the largest producer of sisal and its hardened fiber. The fiber, present in sisal leaves, is mainly used in the manufacture of ropes, strings, and carpets, but this corresponds to approximately 5% of its weight, with all the vegetal saponin-rich residue being discarded. Differently, in several countries, the juice of *Agave sisalana* leaves has great ethnopharmacological importance because it is used as an antiseptic in the topical treatment of skin diseases as well as a poultice on wounds [5,8].

Pomegranate cultivation is currently expanding due to the increase in domestic demand and good expectations for export. Thus, pomegranate peel is also an abundant residue in Brazil, and as it contains numerous phytochemical compounds it has been used for centuries to treat various diseases [9]. In Brazil, especially in the northeast, the bark of pomegranate is popularly used in the treatment of mouth and throat inflammation [10]. In addition, in vitro and in vivo studies with different parts of the plant have demonstrated its anti-microbial and anti-inflammatory proprieties [5,8].

Aiming to develop a new, topical, anti-inflammatory drug from vegetal residues of two saponin-rich species, this paper evaluated extracts developed from sisal and pomegranate residues through in vitro phytochemical, pharmacological, and toxicological studies; the extracts were analyzed both isolated and in an association. In an unprecedented way, a mucoadhesive gel containing the extracts in association was also analyzed.

The development of the gel with mucoadhesive properties is related to its greater ability to adhere to the skin or mucosa, prolonging the contact time and effect of the plant active ingredients of sisal and pomegranate extracts at the site of action [11]. Therefore, the development of an alternative approach, such as the use of hydrogel polymers loaded with natural anti-inflammatories, can accelerate wound healing and achieve tissue regeneration.

In the literature, the combined use of different polymers loaded with active chemical substances has received great attention from researchers, as these can accelerate wound healing and achieve tissue regeneration [12,13,14,15]. Polomocihydroxybutyrate (PHB), a natural hydrophobic polymer, when associated with another polymer (co-electrofoliation), such as gelatin [13], chitosan [14] or poly(ε-caprolactone) [15], can be used as a biomaterial for wound dressings. For example, El-Shanshory [13] reported that a metronidazole immobilized PHB/gelatin nanofibrous scaffold accelerated secondary intention wound healing.

## 2. Results and Discussion

### 2.1. Saponins

As presented in Table 1, in the analysis of the isolated extracts, the presence of saponins was observed in RR, RS, A, G1, and G2.

Identifying the health benefits of phytochemicals is an essential step in the development of pharmaceuticals and functional foods. In this context, screening for saponins, secondary metabolites used in the synthesis of corticosteroids, is of paramount importance for the proposed residual reuse in this study. The goal was the formulation of a topical gel for potential use on the oral mucosa in patients suffering from inflammation in this region [16,17].

In this study, the concentration of saponins found in the sisal residue extract (RS) was 29.91 g of saponins per 100 g of dry extract. As for the presence of saponins in pomegranate peel extract, this study revealed an average value of 15.83 g/100 g. In the literature, there are no comparative data, which underscores the uniqueness of this research. The combination of sisal and pomegranate residue extracts (A) resulted in a saponin concentration of 22.99 g per 100 g of dry extract. This value is close to the sum of the saponin concentrations present in 50 g of each extract, which would be 22.86 g.

The gel containing the combination of extracts (G2), both at 0.6%, exhibited a saponin concentration of 0.52 g per 100 g of gel, meaning a higher saponin concentration when compared to the simple summation of values of this metabolite in the isolated extracts at a concentration of 0.6%, which would be 0.27 g per 100 g of gel. This suggests that the saponin concentration was not compromised by the formulation; on the contrary, it facilitated saponin detection. The association of extracts (G2) showed a concentration of 0.52 g/100 g, lower than the associated extracts, but still higher than those observed in the isolated extracts.

The absence of phytochemical studies on sisal and pomegranate peel, both individually and in combination, limits a more in-depth discussion of the saponin data obtained. However, these findings already indicate that high saponin concentrations are likely responsible for the pharmacological activities analyzed in this study. This is because saponins exhibit anti-inflammatory activities and are used in medicinal chemistry for the production of steroidal anti-inflammatory drugs [16,17,18].

### 2.2. Cellular Viability

**Figure 1:**
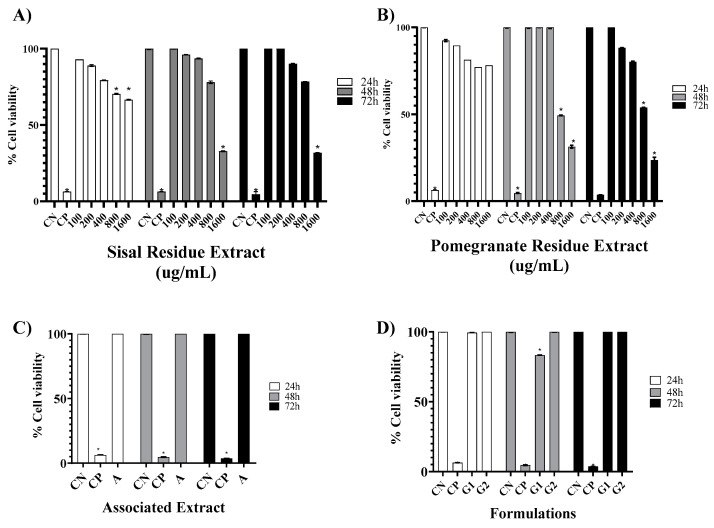
Mean ± SD of the values in % referring to the cell viability rate in NIH 3T3 fibroblasts at different evaluation times (24, 48, and 72 h) after the following treatments: saline—negative control (NC); 2% Tween 80—positive control (PC); isolated extracts of sisal (SR) and pomegranate (PR) waste ((**A**,**B**), respectively) at concentrations of 100, 200, 400, 800, and 1600 µg/mL; associated extract containing SR and PR, each at concentration of 600 µg/mL (**C**); and formulations G1 (base) and G2 containing SR and PR at concentrations of 600 µg/mL (**D**). *p* < 0.05 means a significant difference compared to the PC group. One-way ANOVA followed by Tukey’s post hoc test. The asterisk (*) indicates a significant difference (*p* < 0.05) between groups.

Figure 1A depicts the cell viability exhibited exposure to SR at the time intervals of 24, 48, and 72 h. Concentrations of 100 µg/mL (92.95 ± 0.03%, 109.46 ± 0.31%, and 130.14 ± 0.28%) and 200 µg/mL (88.90 ± 0.63%, 96.06 ± 0.31%, and 114.90 ± 0.28%) promoted increasing cell viability over time. The treatments and positive control (PC) showed significant differences compared to the negative control (NC), *p* < 0.05. Except for the concentration of 1600 µg/mL (66.56 ± 0.31%, 32.86 ± 0.31%, and 31.76 ± 0.13%), all of the tested concentrations exhibited cell viabilities exceeding 70%.

The cell viability following exposure to PR at the time intervals of 24, 48, and 72 h is shown in Figure 1B. Similar to RS, the treatments and positive control (PC) exhibited significant differences compared to the negative control (NC), *p* < 0.05. Furthermore, it can be observed that concentrations of 800 µg/mL and 1600 µg/mL, at 48 and 72 h, displayed cell viabilities below 70% (49.31 ± 0.19%, 53.91 ± 0.19%, and 31.30 ± 0.81%, 23.65 ± 1.55%, respectively), thus not promoting fibroblastic proliferation.

In the MTT assay, through linear regression, the cytotoxicity concentration 50% (CC50%) values for the isolated extracts at 72 h were established. The CC50% went to 3757.00 µg/mL for SR and 2064.91 µg/mL for PR.

Figure 1C presents the results obtained for the association of extracts (A) at the time intervals of 24, 48, and 72 h at a concentration of 600 µg/mL for both extracts (116.93 ± 0.05%, 209.61 ± 0.05%, and 249.79 ± 0.02%, respectively). Significant differences were found for the treatment and the PC compared to the NC (*p* < 0.05). Moreover, the cell proliferation rate was higher than the NC (100.00 ± 0.13%, 147.32 ± 0.44%, and 148.12 ± 4.96%), demonstrating that A is a potent fibroblastic cell-cycle stimulator.

Figure 1D displays the cell viability rate resulting from treatment with the base gel (G1) and the gel containing the association of extracts (G2). Both formulations showed significant differences compared to the NC (*p* < 0.05). Formulation G1 exhibited a cell viability above 70% at 24, 48, and 72 h (99.45 ± 0.11%, 83.29 ± 0.21%, and 118.58 ± 0.16%, respectively). For G2, the cell viability increased at the analyzed time intervals (116.93 ± 0.05% at 24 h, 183.94 ± 0.32% at 48 h, and 187.10 ± 0.11% at 72 h), as well as surpassing that of the NC (100.00 ± 0.13% at 24 h, 147.32 ± 0.44% at 48 h, 148.12 ± 4.96% at 72 h).

To evaluate the cytotoxic effect caused by the extracts of sisal and pomegranate waste (SR and PR), as well as the associated extracts, the base gel, and the gel with the association, the MTT assay was chosen. According to the parameters used by to classify the degree of toxicity, the cytotoxicity presented by SR and PR in 24 h was considered to be of little importance, because the CC50% values obtained confirmed that the concentrations analyzed were not able to reduce cell viability by 50%; the association of extracts showed high cell viability; the formulation of the base gel proved to be free of cytotoxicity; and the formulation of the gel with the association increased cell viability with increased time [13].

The results obtained in the MTT assay showed that all SR concentrations increased cell viability as a function of treatment time. In complement, Dunder et al. [7] in the same test at concentrations of 50–500 µM of hexanic fraction of sisal did not induce mortality in the same cell lineage of this study. In contrast, Favato [19] with the concentration of 50 µg/mL of sisal acid hydrolysis extract demonstrated satisfactory results of cytotoxicity to melanoma cells and low systemic genotoxicity, decreasing the risk of the emergence of neoplasms secondary to treatment [19]. Texeira [20] reported that at the concentration of 400 μg/mL, the extract of sisal acid hydrolysis exerted cytotoxicity, having promoted the death of 42.73% of small lung cancer cells. Thus, in this study, SR was shown to be free of toxicity to 3T3 fibroblasts and from the literature reports, future analysis of SR antitumor activity should be directed to tumor cell lines.

For PR, the concentration of 200 μg/mL, at the three analyzed times, did not show cytotoxicity, unlike the other concentrations, in which the concentration of 1600 μg/mL showed less than 50% viability in 72 h. Lorenzoni [21] also reported increased cell viability in a 3T3 fibroblast cell line as non citotoxic. The cytotoxicity exerted at the highest concentrations of the PR may be related to the fact that it is in glycol solution.

The association of plant secondary metabolites is of interest for the potentiation of their pharmacological effects, but the cytotoxicity of the association must be analyzed. The association of SR and PR did not impair cell viability; on the contrary, viability increased as a function of time. In the literature, there are no reports on the cytotoxicity of the association of SR and PR, but after analyzing other associations, it was found in the study of Botan [22] that the association of *Morus nigra*, *Ziziphus joazeiro*, and *Vitis vinifera* increased cell viability within 24 h in a RAW 264 macrophage lineage. In summary, the association of bioactive plant compounds shows promise.

The cytotoxicity analysis for in vitro formulations have been shown to be an alternative to toxicological tests on animals. Thus, the analysis of G1 and G2 was also performed using the MTT test. The results showed that G1 and G2 showed no cytotoxicity over the three treatment times. Additionally, in vitro studies on formulations of natural extracts were not found, further highlighting the originality of this research.

The absence of cytotoxicity made it possible to proceed with anti-inflammatory studies. Macrophages act as the first line of defense, then, the inflammatory cascade. Macrophages are cells derived from monocytes. These are released into the bloodstream after their formation in the bone marrow and subsequently exit the blood to differentiate into macrophages in the tissues. Macrophages are found in all connective tissue sites, and in addition, they are concentrated in various organs such as the liver, spleen, and lymph nodes, where they are related to the body’s defenses. Thus, they play a key role in innate immunity, effectively participating in inflammatory mechanisms and modulation of the immune system [23,24,25].

### 2.3. Phagocytosis

**Figure 2:**
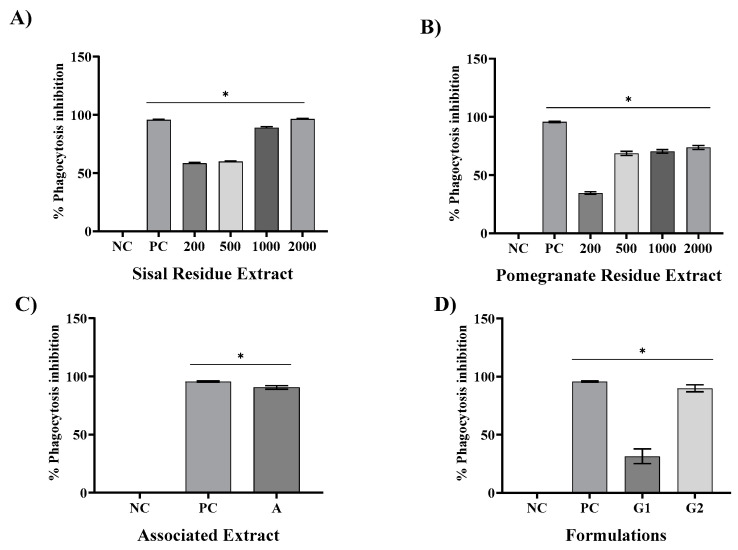
Mean ± SD of the values in % regarding the inhibition of phagocytosis in RAW 264 macrophage after the following treatments: saline—negative control (NC); dexamethasone 40 µg/mL—positive control (PC); isolated extracts of sisal (SR) and pomegranate (PR) waste ((**A**,**B**), respectively) at concentrations of 200, 500, 1000, and 2000 µg/mL; associated extract containing SR and PR each at a concentration of 600 µg/mL (**C**); and formulations G1 (base) and G2 containing SR and PR each at a concentration of 600 µg/mL (**D**). One-way ANOVA followed by Tukey’s post hoc test. The asterisk (*) indicates a significant difference (*p* < 0.05) between groups.

In Figure 2A, we present the results pertaining to the inhibition of phagocytosis (IP) by SR. These results indicate a progressive inhibition pattern, with values of 58.2%, 59%, 88%, and 96.28% observed at SR concentrations of 200 µg/mL, 500 µg/mL, 1000 µg/mL, and 2000 µg/mL, respectively. In comparison, the positive control (PC), represented by dexamethasone at a concentration of 40 µg/mL, demonstrated a robust inhibition of 95.06%. Notably, all concentrations of PR and the PC exhibited statistically significant differences (*p* < 0.05) when compared to the negative control (saline).

Transitioning to Figure 2B, we elucidate the PI results obtained with the PR extract. These findings reveal a dose-dependent inhibitory effect, with values of 37.5%, 68%, 70%, and 74% achieved at PR concentrations of 200 µg/mL, 500 µg/mL, 1000 µg/mL, and 2000 µg/mL, respectively. In contrast, dexamethasone consistently displayed a robust inhibition of phagocytosis, at 95.06%. Similar to SR, all concentrations of PR and the PC exhibited statistically significant distinctions (*p* < 0.05) from the negative control saline. By considering the 50% concentration inhibition (IC50%), SR exhibited an IC50% of 460 µg/mL, while PR exhibited an IC 50% of 596 µg/mL. For standardization purposes, both SR and PR were set at a concentration of 600 µg/mL. The combination of these extracts (A) resulted in a noteworthy phagocytosis inhibition of 90.54%, closely mirroring the inhibitory effects of SR at 2000 mg/mL (96.28%) and dexamethasone (95.60%). Furthermore, the associated extract and PC demonstrated statistically significant distinctions from the NC at *p* < 0.05, as delineated in Figure 2C. Lastly, Figure 2D illustrates that the inclusion of the associated extracts within a gel formulation (G2) led to a substantial IP, with a value of 89.86%. This gel, in addition to the base gel and the dexamethasone-containing gel (40 µg/mL), all exhibited statistically significant distinctions from the NC (*p* < 0.05).

Phagocytosis begins with macrophage dissemination and morphological change, in addition to the activation of lysosomes and the production of reactive oxygen species. Thus, the phagocyte uses surface receptors to bind to and surround a microorganism, forming a vesicle called a phagosome. The surface receptors also release signals that stimulate the fusion of phagosomes with lysosomes originating from the phagolysosomes and inflammation-activating signals that trigger the respiratory burst and the production of nitric oxide to destroy and eliminate the phagocyted elements as well as the invaders [23].

In this study, SR and PR promoted significant inhibition of phagocytosis in all the analyzed concentrations. However, SR presented a greater ability to inhibit phagocytosis at concentrations of 1000 and 2000 µg/mL. With the association of SR and PR, it was possible to verify the occurrence of a pharmacological synergism that potentiated the anti-inflammatory effect, since the associated extracts, each one at a concentration of 600 µg/mL, promoted phagocytosis inhibition close to that presented by SR at a concentration of 2000 µg/mL. The inclusion of the associated extracts in a gel formulation (G2) promoted phagocytosis inhibition similar to that of thegel containing dexamethasone, a product widely used in medical practice. Silveira [25] found that *Chorisia speciosa* and *Hymenaea courbaril* A. decreased the phagocytic capacity of macrophages by 49.80%. Sansone [26], on the other hand, found that the phagocytic inhibition capacity of soluble non-starch polysaccharides from bananas, at a concentration of 200 µg/mL, was 49.3%.

### 2.4. Macrophage Spreading

**Figure 3:**
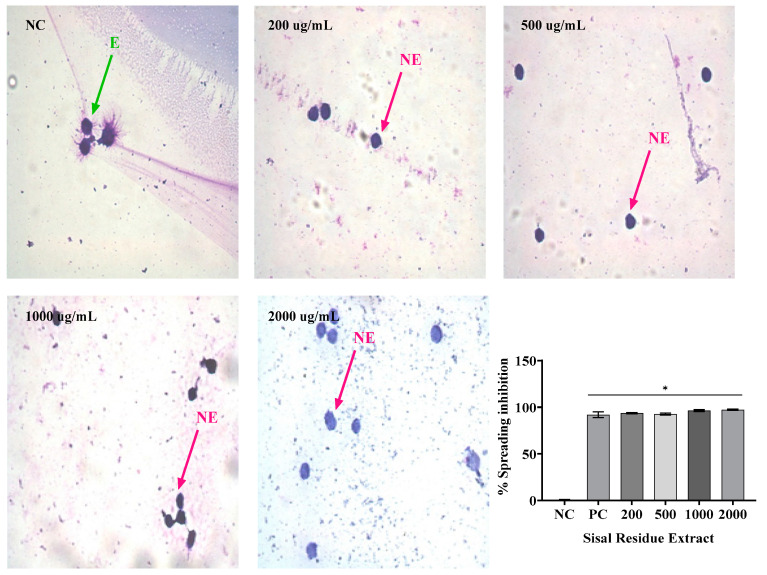
Mean ± SD of the values in % of spreading inhibition in RAW 264 macrophages after the following treatments: saline—negative control (NC); dexamethasone 40 µg/mL—positive control (PC) and isolated extract of sisal residue (SR) at concentrations of 200, 500, 1000, and 2000 µg/mL. *p* < 0.05 means a significant difference compared to the NC group. Also, in Figure 4, the isolated PR promoted the following inhibitions at the different concentrations analyzed: 200 µg/mL—39%, 500 µg/mL—40%, 1000 µg/mL—80%, and 2000 µg/mL—88%. The isolated extract, at all concentrations analyzed and the PC, differed from NC. ANOVA followed by Tukey’s post hoc test. The asterisk (*) indicates a significant difference (*p* < 0.05) between groups.

**Figure 4:**
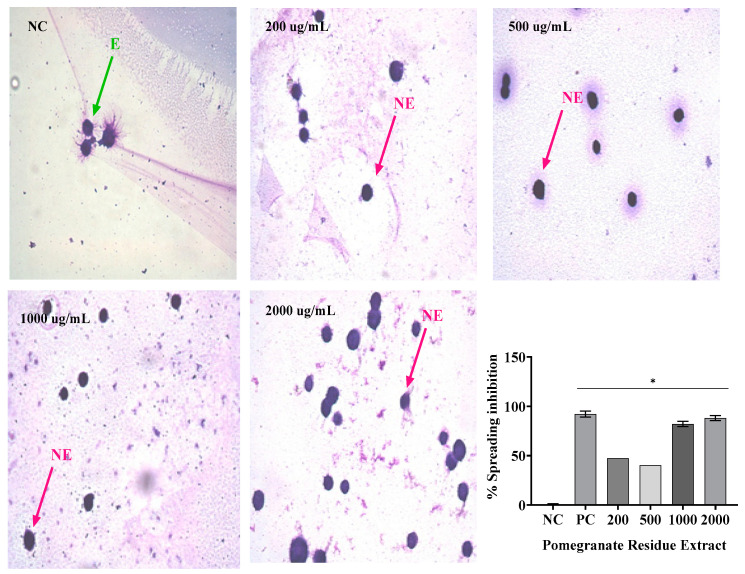
Mean ± SD of the values in % of spreading inhibition in RAW 264 macrophage after the following treatments: saline—negative control (NC); dexamethasone 40 µg/mL—positive control (PC); and isolated extract of pomegranate residue (PR) at concentrations of 200, 500, 1000, and 2000 µg/mL. One-way ANOVA followed by Tukey’s post hoc test. The asterisk (*) indicates a significant difference (*p* < 0.05) between groups.

**Figure 5:**
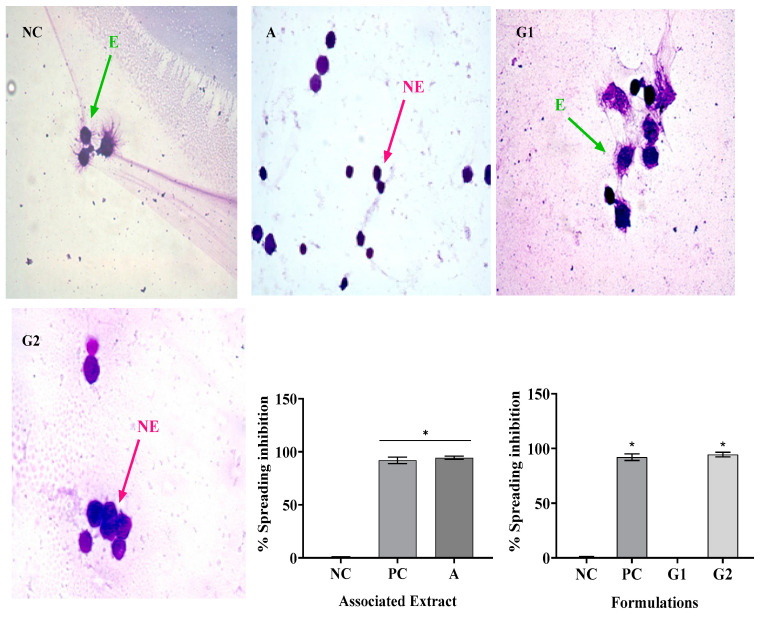
Mean ± SD of the % spreading inhibition values in RAW 264 macrophages after the following treatments: saline—negative control (NC); dexamethasone 40 µg/mL—positive control (PC); associated extract containing extracts of sisal (SR) and pomegranate (PR) waste, each at 600 µg/mL; and formulations G1 (base) and G2, containing SR and PR at 600 µg/mL concentrations. *p* < 0.05 means a significant difference compared to the NC group. One-way ANOVA followed by Tukey’s post hoc test. The asterisk (*) indicates a significant difference (*p* < 0.05) between groups.

The results presented in Figure 3, Figure 4 and Figure 5 demonstrate the inhibitory effects of the treatments on macrophage spreading, with SR and PR extracts being the primary focus. In Figure 3, when cells were spreading on the negative control (NC) and subsequently treated with SR, it was observed that SR, when administered alone, induced significant inhibitions at various concentrations: 200 µg/mL—93%, 500 µg/mL—92%, 1000 µg/mL—96%, and 2000 µg/mL—97.5%. Concurrently, the positive control (PC) exhibited a comparable inhibitory effect, with a 92.03% reduction in cell spreading. Importantly, the SR extract at all concentrations examined, as well as the PC, displayed statistically significant differences (*p* < 0.05) compared to the NC. In Figure 4, the same analytical approach was applied to both the SR and PR treatments. SR, when administered alone, showed similar inhibitory effects across different concentrations: 200 µg/mL—93%, 500 µg/mL—92%, 1000 µg/mL—96%, and 2000 µg/mL—97.5%. Notably, the PC demonstrated an equivalent inhibitory effect, reducing cell spreading by 92.03%. When PR was administered, it exhibited its own distinct inhibitory pattern: 200 µg/mL—39%, 500 µg/mL—40%, 1000 µg/mL—80%, and 2000 µg/mL—88%. Again, the PC achieved a similar level of inhibition. Both extracts, across all concentrations tested, along with the PC, demonstrated statistically significant distinctions (*p* < 0.05) compared to the NC.

In Figure 5, the focus shifts to the effects of the associated extracts and gel formulations on cell spreading. The associated extract showed a significant inhibition of 92%, while G2, a gel formulation containing the associated extracts, inhibited cell spreading by 94%. In contrast, G1, a different gel formulation, and the NC showed no significant inhibition. Both the associated extracts and G2 differed significantly from the NC (*p* < 0.05). In summary, Figure 3, Figure 4 and Figure 5 collectively demonstrate the efficacy of the SR and PR extracts in inhibiting cell spiking and spreading, with similar inhibitory patterns observed for SR and PC. Additionally, the combination of the extracts in the G2 formulation displayed a pronounced inhibitory effect on cell spreading, emphasizing the potential therapeutic relevance of this treatment.

Not only can phagocytosis be evaluated in vitro directly by the ability to visualize phagocytes engulfing particles or microorganisms, but it can also be evaluated indirectly by the frustrated attempt at phagocytosis, manifested by macrophage adherence and spreading on glass coverslips [24]. Thus, the in vitro evaluation of the anti-inflammatory activity using the spreading technique of macrophages was performed, because macrophages adhere to the glass when stimulated, becoming spiked. In addition, in the presence of anti-inflammatory compounds, macrophage spreading on slides is reduced [25].

In this study, treatment with SR at the four concentrations analyzed resulted in macrophage spreading inhibition values similar to dexamethasone, while PR promoted inhibition similar to dexamethasone only at its highest concentration (2000 µg/mL). The association of SR and PR, as well as the gel containing the extracts, promoted similar inhibition to the gel containing dexamethasone. PR did not impair the ability of SR to inhibit macrophage spreading and the association showed effective anti-inflammatory activity. In this regard, Ota et al. [26] nalyzed macrophage spreading with four doses of garlic hydroalcoholic extract; doses II to IV showed 100% macrophage spreading, unlike our study [26].

### 2.5. Lysosomal Stabilization

**Figure 6:**
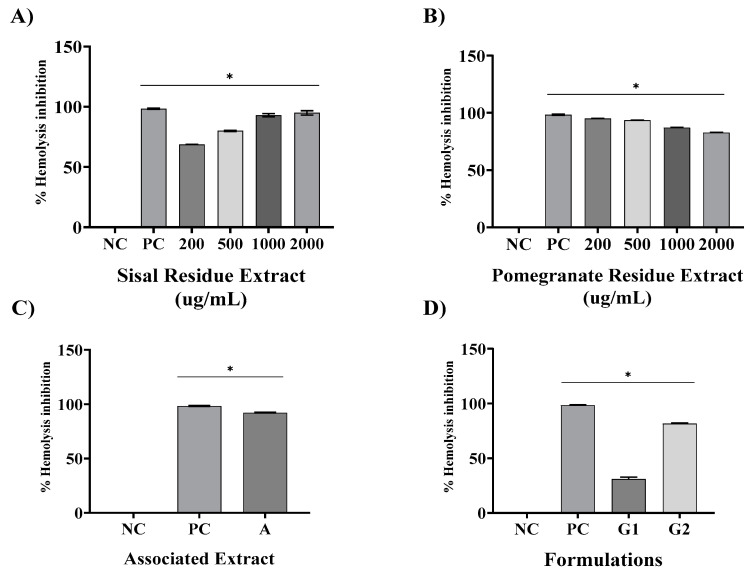
Mean ± SD of the values in % of hemolysis inhibition after the following treatments: saline—negative control (NC); dexamethasone 100 µg/mL—positive control (PC); isolated extracts of sisal (SR) and pomegranate (PR) waste ((**A**,**B**), respectively) at concentrations of 200, 500, 1000, and 2000 µg/mL; associated extract containing SR and PR each at a concentration of 600 µg/mL (**C**); and formulations G1 (base) and G2 containing SR and PR each at a concentration of 600 µg/mL (**D**). One-way ANOVA followed by Tukey’s post hoc test. The asterisk (*) indicates a significant difference (*p* < 0.05) between groups.

In this analysis, Figure 6A shows that the PC at the concentration of 40 µg/mL promoted 98.39% protection against hemolysis. The treatments with SR promoted protection at different concentrations: 200 µg/mL—68.75%, 500 µg/mL—76.99%, 1000 µg/mL—93.22%, and 2000 µg/mL—95.63%. The PR treatments, shown in Figure 6B, also demonstrated anti-hemolytic activity at concentrations of 200 µg/mL—95.13%, 500 µg/mL—93.61%, 1000 µg/mL—87.19%, and 2000 µg/mL—82.84%. For both extracts analyzed at all concentrations, and for the PC, there was a significant difference (*p* < 0.05) when compared to the NC.

Figure 6C shows that the results obtained with the associated extracts were able to inhibit 95.02% of hemolysis, differing significantly from the negative control (*p* < 0.05). In Figure 6C, it is possible to see that G1 showed 31% protection, not differing from the NC, while G2 conferred 81.64% protection, differing significantly from the negative control (*p* < 0.05). The PC differed from the saline NC significantly, *p* < 0.05.

Another anti-inflammatory evaluation used was the erythrocyte membrane stabilization method. The erythrocyte membrane stabilization method is widely used for the analysis of the anti-inflammatory activity of plant extracts [26]. This is because during the inflammatory process, lysosome membranes are lysed and anti-inflammatory drugs inhibit this process. The membrane of human red blood cells was analyzed in this test because it is similar to the membrane of lysosomes, which in hypotonic medium undergo lysis, similar to what occurs in the inflammatory process. Thus, the inhibition of hypotonicity and lysis of the red blood cell membrane induced by the hypotonic solution was verified as a mechanism of anti-inflammatory activity [27].

Using the same method, it was found that the extract of *Solanum paniculatum* L. at all extract concentrations (15, 30, and 60 μg/mL), significantly protected RBCs against erythrocyte membrane rupture [27]. In addition, Nagaharika et al. [28] observed that at the concentration of 100 µg/mL/mL the alcoholic plant extract of *Jatropha gossypifolia* L. inhibited hemolysis by 56.8%. *C. cujete* leaves and stem also showed great potential for membrane stabilization, a concentration of 100 µg/mL inhibited by 78.81% [29].

Of particular importance is the examination of combined extracts in our study, as exemplified in all assays. The observed synergy resulting from the combination of these extracts yielded a remarkable effect, surpassing the individual effects of the SR and PR extracts. This observation underscores the potential utility of merging these botanical extracts in therapeutic interventions aimed at addressing inflammation-associated conditions. In contrast, our formulations revealed that G1, a specific formulation, exhibited limited pharmacological effects, comparable to the negative control (NC). However, G2, a distinct formulation incorporating the combined extracts, demonstrated a significant anti-inflammatory effect and potential.

## 3. Conclusions

Notably, the gel containing the combined extracts showed significant anti-inflammatory activity and an absence of cytotoxicity, suggesting a promising avenue for further investigations into its therapeutic application in the field of inflammatory disorders. In this direction, in vivo pharmacological and toxicological studies, phytochemical studies (FTIR and HPLC), and formulation quality control analysis are being conducted in order to complete pre-clinical trials of the formulation.

## 4. Materials and Methods

### 4.1. Plant Material

The use of sisal and pomegranate waste has already been regulated by SisGen, through the protocol of the CAPES Print Unesp Project: Exploring Multidisciplinary Approaches for the Development of Phytotherapeutic Products. Registration number: A1266B4. 

The sisal residue (mucilage) originated from Valente city, Bahia state. The species under study is *Agave sisalana*; the specimen belongs to the Herbarium Assisense (HASSI) of the São Paulo State University—UNESP (Assis, state of São Paulo), where a voucher specimen is deposited under the number 2597.

The pomegranate peel was acquired from Companhia Santos Flora, Mairiporã-SP. The peel comprised a single batch already shredded, dehydrated, and sterilized.

### 4.2. Extract Preparation 

The sisal extract preparation with the mucilage, composed of solid and liquid residues, results from fiber extraction from sisal leaves. After being pressed and filtered, it produces a liquid known as sisal juice. The sisal residue extract (SR) was prepared according to the following method: (1) acid hydrolysis with hydrochloric acid 30% (Synth, SP, Brazil), heated at 120 °C for 60 min; (2) solution centrifugation at 4000 rpm for 20 min to obtain a residue; (3) precipitate neutralization with sodium hydroxide 5% (Exodus, SP, Brazil), followed by (4) precipitate lyophilization. From the dried precipitate, 10 g were dissolved in 100 mL of 5% sodium hydroxide (Anidrol, Diadema, SP, Brazil). The solution was stirred for 1 h and filtered (Fisatom, model 803, São Paulo, SP, Brazil). (6) The liquid fraction was neutralized with 0.1 molar acetic acid (Synth, SP, Brazil). The filtered liquid fraction extract obtained was lyophilized and stored at 4 °C. 

To prepare the pomegranate residue extract (PR) the following steps were applied: (1) maceration of 10 g of pomegranate peel powder with 30 mL of ethanol at 70% for 24 h, followed by filtration. This process was repeated five times using the residual solids from each filtration until no soluble solids could be observed in the extract. (2) The volumes obtained from all five extractions were mixed and concentrated in a rotary evaporator at 60 °C to obtain the soft extract. (3) The soft extract was lyophilized and prepared in a 30:70 (p/v) proportion with propyleneglycol PA (Synth, SP, Brazil). This process was followed by an agitated water bath, at 40 °C, until complete homogenization. 

### 4.3. Association of Sisal and Pomegranate Residue Extracts

To potentiate the isolated effects of the sisal and pomegranate residue extracts, they were associated (A). The concentrations of each extract used in the association corresponded to the effective concentration 50 (CC50%) values obtained in the phagocytosis assay, and corresponded to the values of 600 µg/mL of the SR and 600 µg/mL of the PR.

### 4.4. Preparation of the Mucoadhesive Gel

To prepare the formulation, the following were used: carboxymethyl sodium (Exodus, SP, Brazil) at 1.6% (*w*/*w*), glycerin (Synth, SP, Brazil) 1.2% (*w*/*w*), methylparaben (Synth, SP, Brazil) at 0.1% (*w*/*w*), and triethanolamine (Sigma, SP, Brazil) 1.4% (*w*/*w*) as a pH corrector. During the preparation, all of the reagents were dissolved in water and the solution was stirred until a gel-form formulation was obtained. The formulation remained at rest for 24 h. After this time, the formulation was stirred again, and the association of pomegranate and sisal extracts was added at concentrations of 600 µg/g of the SR and 600 µg/g of the PR.

### 4.5. Determination of Total Saponin

The saponin content was measured in a test tube. A volume of 0.25 mL of isolated extract (SR or PR) was pipetted at concentrations of 200, 500, 1000, and 2000 ug/mL; combined extract (A) 600 ug/mL of SR and PR; base gel (G1) PA; and association gel (G2) 600 ug/g of SR and PR), with 0.25 mL of vanillin 10% (*w*/*v*) fresh, and 2.5 mL H_2_SO_4_ 72% (at a cold temperature). The mixture was heated in a water bath for 10 min at 60 °C and cooled. The absorbance of the mixture was measured at a wavelength of 544 nm. Saponin Equijala (Sigma Aldrich, CAS 8047152, Burlington, MA, United States.) was used as the reference standard. The results of the isolated extracts were expressed considering the concentration of saponins per 100 g of dry extract, and for the associated extracts the result considered the concentration of saponins for 50 of each dry extract, that is, 100 g in total. 

### 4.6. Determination of In Vitro Toxicity Using the MTT Assay [3-(4,5-dimethylthiazol-2-yl)-2,5-diphenyltetrazolium bromide]

The MTT cytotoxicity assay was carried out as described previously by Fracasso, with some modifications. For this essay, mouse fibroblasts of dermal origin (NIH/3T3, ATCC^®^ CRL-1658™) were inoculated in 96-well microtiter plates and incubated in a culture medium for a period of 24 h at 37 °C, 5% CO_2_. After a confluence of approximately 75% (24 h), these cells were exposed to five different concentrations of SR and PR (100 µg/mL, 200 µg/mL, 400 µg/mL, 600 µg/mL, and 1600 µg/mL), A (600 µg/mL of SR and 600 µg/mL of PR), G1, and G2 (600 µg/mL of SR and 600 µg/mL of PR), to the negative control (physiological solution). and the positive control (2% (*v*/*v*) Tween 80). The treatment times were 24 h, 48 h, and 72 h. The five concentrations were chosen to calculate the cytotoxicity concentration of the isolated extracts (CC50%) in 24 h.

### 4.7. Evaluation of Anti-Inflammatory Activity In Vitro

#### 4.7.1. Experimental Design

To evaluate the anti-inflammatory activity of sisal (SR) and pomegranate (PR) extracts, four concentrations were used (200 µg/mL, 500 µg/mL, 1000 µg/mL, and 2000 µg/mL). 

To evaluate the anti-inflammatory activity of the association of sisal and pomegranate residue extracts (A), concentrations of 600 µg/mL of SR and 600 µg/mL of PR were used. The concentrations used in the formulation of the gel (G2) containing the association of sisal and pomegranate extracts were 600 µg/g of SR and 600 µg/g of PR. Dexamethasone (100 μg/gr) was used as a positive control. Saline solution (0.9% saline) and base gel (G) were used as negative controls. 

#### 4.7.2. Cell Culture

Murine macrophages of the 264.7 RAW (ATCC TIB-71) strain were thawed and cultivated in a cell culture flask with Dulbecco’s Modified Eagle Medium (DMEM) Ham’s F-12 culture medium at 37 °C, 5% CO_2_. The anti-inflammatory efficacy and cytotoxicity assays were performed when the culture reached about 70–80% confluence.

#### 4.7.3. Selection of Macrophages

Cells were harvested using a cell scraper, counted in a Neubauer chamber, and centrifuged at 1500 rpm for 5 min. Then, the supernatant was discarded, and the cells were resuspended in a culture medium to reach the desired concentration for each experiment.

#### 4.7.4. Phagocytosis 

The assay was performed following the method described by Fracasso with a few modifications. Once the slides were ready, they were read in triplicate using an optical microscope at 400× magnification, performing a total count of 100 cells. 

The inhibition of phagocytosis (IP) was calculated using the following formula:

(1) $$\mathrm{IP}\;(\%)=\frac{E_{0}-E_{T}}{E_{0}}\times 100$$

where *E*_0_ represents the mean value of the number of cells in the negative control group that phagocytosed the zymosan particles; and *E_T_* represents the mean value of the number of cells of the treated group that phagocytosed the zymosan particles.

#### 4.7.5. Macrophage Spreading

For the macrophage spreading assay, the method described by Fracasso et al. [6] was used. Once the slides were ready, there were read triplicates under an optical microscope at 400× magnification, performing a total count of 100 cells. 

The inhibition of spreading (IS) was calculated using the following formula:

(2) $$\mathrm{IS}\;(\%)=\frac{E_{0}-E_{T}}{E_{0}}\times 100$$

where *E*_0_ represents the mean value of the number of spread cells in the negative control group; and *E_T_* represents the mean value of the number of cells spread in the treated group.

#### 4.7.6. Membrane Stabilization 

For the evaluation of the human red blood cell (HRBC) membrane stabilization, the method proposed by Ananthi and Chitra [26] was employed, with some modifications. The test reaction was performed with the addition of 2 mL of hyposaline solution (0.18%) to induce hemolysis, 1 mL of sodium phosphate buffer (0.1 M, pH 7.4), 1 mL of the analyzed samples, and 0.5 mL of HRBC solution. The hemoglobin content in the suspension was estimated using a spectrophotometer at 560 nm.

The percentage of protection can, hence, be calculated from the equation given below:

(3) $$\mathrm{Protection}\;(\%)=\frac{E_{0}-E_{T}}{E_{0}}\times 100$$

where *E*_0_ represents the mean absorbance value of the negative control group; and *E_T_* represents the mean absorbance value in the treated group.

### 4.8. Statistical Analysis

The data are expressed in terms of mean ± standard deviation. Statistical analysis was performed using GraphPad Prism version 7. To verify the statistical differences between the groups, a one-way analysis of variance (ANOVA) is performed according to the experimental protocol, followed by Tukey’s multiple comparison test. For all analyses, a *p*-value of <0.05 was considered significant.

## Figures and Tables

**Table 1:** Dosage in grams of total saponins present in 100 g of dry extract of PR and SR, extracts in association (A), and formulations G1 and G2 in 100 g of the gel.

Extract/Formulation	Sample	Total Saponins (g/100 g)
Extract	PR	15.83 ± 0.93
Extract	SR	29.91 ± 0.33
Extract	A	22.99 ± 0.01
Formulation	G1	0.00 ± 0.00
Formulation	G2	0.52 ± 0.01

## Data Availability

Data are contained within the article.
